# An Isolated and Deeply Divergent *Hynobius* Species from Fujian, China

**DOI:** 10.3390/ani13101661

**Published:** 2023-05-17

**Authors:** Zhenqi Wang, Siti N. Othman, Zhixin Qiu, Yiqiu Lu, Vishal Kumar Prasad, Yuran Dong, Chang-Hu Lu, Amaël Borzée

**Affiliations:** 1 The Co-Innovation Center for Sustainable Forestry in Southern China, College of Biology and the Environment, Nanjing Forestry University, Nanjing 210037, China; 2 Laboratory of Animal Behaviour and Conservation, College of Biology and the Environment, Nanjing Forestry University, Nanjing 210037, China; 3 Jiangsu Agricultural Biodiversity Cultivation and Utilization Research Center, Nanjing 210014, China

**Keywords:** Hynobidae, species description, bamboo forest

## Abstract

**Simple Summary:**

What does not have a name is difficult to understand and protect. Upon the unexpected discovery of an *Hynobius* salamander in Fujian province, China, we worked on understanding its relationship with other species and ultimately describing it. Please welcome the Fujian Bamboo Salamander to science, a segregated species based on genetics and morphology. While it is related to other southern mainland Chinese species, it may have diverged earlier and share some similarities with morphology and behavior with the Anji salamander. The Fujian Bamboo Salamander is special as it produces vocalization when under threat. The species is, however, incredibly rare, fitting the definition of Critically Endangered in the IUCN Red List of Threatened Species.

**Abstract:**

It is important to describe lineages before they go extinct, as we can only protect what we know. This is especially important in the case of microendemic species likely to be relict populations, such as *Hynobius* salamanders in southern China. Here, we unexpectedly sampled *Hynobius* individuals in Fujian province, China, and then worked on determining their taxonomic status. We describe *Hynobius bambusicolus* sp. nov. based on molecular and morphological data. The lineage is deeply divergent and clusters with the other southern Chinese *Hynobius* species based on the concatenated mtDNA gene fragments (>1500 bp), being the sister group to *H. amjiensis* based on the *COI* gene fragment, despite their geographic distance. In terms of morphology, the species can be identified through discrete characters enabling identification in the field by eye, an unusual convenience in *Hynobius* species. In addition, we noted some interesting life history traits in the species, such as vocalization and cannibalism. The species is likely to be incredibly rare, over a massively restricted distribution, fitting the definition of Critically Endangered following several lines of criteria and categories of the IUCN Red List of Threatened Species.

## 1. Introduction

Anthropogenisation of landscapes, and other human activities, have brought the world to the sixth great mass extinction [1,2]. The threats to species are not equal, and large-bodied species [3], as well as species with narrow spatial ranges [4], are principally impacted. The toll on species is staggering [5], and between 900 and 130,000 species have become extinct since the 1500s (www.iucnredlist.org/statistics; accessed on 3 May 2023). Similarly, species that have not yet been described are going extinct before being documented [6] and without known impacts [7] (as seen, for instance, in spiders [8]). Biodiversity loss is close to a tipping point [9], and conservation actions are the last tool to maintain evolutionary patterns free of anthropomorphic selection [10]. However, for conservation to be achieved, practitioners need to know what to protect, and thus species need to be described currently, conservation does not correspond to threat status [11], and we can only protect what we know.

With 41% of species listened as threatened by the IUCN Red List of Species (www.iucnredlist.org; accessed on 3 May 2023), amphibians are the most threatened animal class, with habitat loss being one of the principal drivers of species decline [12,13]. For the status of amphibians to improve, a clear taxonomy is first needed, and despite the large number of species described in China every year [14], many species are still in need of formal description or taxonomic revision. This need is especially true for southern China, a hotspot for biodiscovery [15] and conservation needs [16].

All *Hynobius* salamanders species were expected to have been described in China, although the taxonomic resolution of the genus is still an ongoing work, with some recent descriptions in other range countries of the genus, including the Republic of Korea [17] and Japan [18,19,20]. There are five described *Hynobius* species in mainland China, all part of the Southern Chinese group and all meant to be breeding in lentic water bodies, and four species on Taiwan Island, belonging to a segregated phylogenetic group and breeding in small cool mountain streams [21]. All these species are terrestrial, partially fossorial, and breed through larval development in water bodies. The species in southern China belong to the *Hynobius chinensis* group and include *H. chinensis* Günther, 1889 [22], *H. amjiensis* Gu, 1992 [23], *H. guabangshanensis* Shen, 2004 [24], *H. maoershanensis* Zhou, Jiang and Jiang, 2006 [25] and *H. yiwuensis* Cai, 1985 [26].

A *Hynobius* salamander was reported from Fujian province in 1978 and identified as *H. chinensis* [27]. However, following the multiple species descriptions since then, the distribution of *H. chinensis* is now known to be restricted to the Hubei province, and the morphological differences between the species have been clarified, including embryos [28]. The *Hynobius* salamander collected in 1978 from Fujian is therefore considered an undescribed species, and no additional individual has been found in the area since then, potentially resulting from local extirpations. As a result, upon encountering *Hynobius* salamanders in Fujian, we tested for phylogenetic clustering within the *H. chinensis* clade, then proceeded to determine their taxonomic status with phylogenetic tools, and accordingly described a new species with specific morphological characters.

## 2. Materials and Methods

### 2.1. Field Sampling

We found two individuals of the genus *Hynobius*, one in January and one in August 2022, from Quxi village, Liancheng County, Fujian, China (25.566° N, 116.938° E). We do not provide precise GPS coordinates here to protect the species from harvesting for the pet trade. The two individuals were found in a bamboo forest (*Phyllostachys* cf. *edulis*) about 1500 m above sea level. As ten days (24–26 January and 4–10 August) of fieldwork during adequate sampling seasons and times of day resulted in only two adult individuals, and the population size at this location, and likely for this species, is likely lower than 200 individuals, we followed the IUCN recommendation on ethical sampling [29]. We did not collect the individuals but instead orally swabbed them to obtain genetic materials and measured them (see Section 2.5 Morphometry) before releasing them at the point of capture to avoid a further threat to the species. In addition, we observed two waterbodies with eight pairs of egg sacs in January 2022, and we collected one egg sac deposited by the species for morphological measurement and to study the development of larvae in the species before releasing them at the point of capture. We used the entire body of an embryo from the egg sacks collected that died at an early developmental stage to extract DNA for a third individual (ID: 22HyF007). We consider this individual unlikely to be related to the adults swabbed due to the distance between the egg sacks and the adults in view of the dispersal abilities of the genus [30].

### 2.2. Ethical Approval

All applicable international, national, and/or institutional guidelines for the care and use of animals were strictly followed. All animal sample collection protocols complied with the current laws of the People’s Republic of China. All observations and experiments conducted in this study are in agreement with the ethical recommendations of the College of Biology and the Environment at Nanjing Forestry University (IACUC approval number 2022014). We did not collect adult individuals as voucher specimens, in line with the IUCN recommendation on research involving species at risk of extinction [29]. Instead, we relied on one of the individuals raised from the egg mass.

### 2.3. DNA Preparation

We extracted the total genomic DNA for all three samples using the Qiagen DNeasy Blood & Tissue kit (QIAGEN Group, Hilden, Germany) according to the manufacturer’s protocol. We then performed standard Polymerase Chain Reaction (PCR) amplifications for all 15 samples in 20 µL of total reaction per tube, containing 35 to 50 ng/μL of template DNA (Table 1). The final concentrations of the other PCR reagents were such as 0.125 μM for each forward and reversed primer, 1× Ex taq Buffer (Takara; Shiga, Japan), 0.2 mM of dNTPs Mix (Takara; Shiga, Japan), 1.875 mM of magnesium chloride (MgCl_2_), 0.1 unit/μL of Ex taq (HR001A, Takara; Shiga, Japan), and double distilled water added to make up the final volume. The PCR thermal profiles for each primer fragment are described in Table 1.

### 2.4. Molecular Analyses

We trimmed all DNA sequences of the three gene fragments, isolated from the three *Hynobius* individuals, and aligned them with their most homologous sequences retrieved from Genbank (see accession numbers and references in Supplementary Table S1). To test the phylogenetic relationship of East Asian Hynobid salamanders, we verified the monophyly of the candidate species and made sure it is not an introduced population of any of the other species; we reconstructed Bayesian Inference (BI) trees for four independent datasets: (i) 16S rRNA gene (*n* sequence = 97, length = 528 bp), (ii) protein-coding Cytochrome b (Cyt*b*) gene (*n* sequence = 117, length = 630 bp), (iii) protein-coding cytochrome c oxidase subunit I (*COI*) gene (*n* sequence = 84, length = 567 bp), and (iv) concatenated 16S rRNA, Cyt*b* and *COI* (*n* sequence = 29, length = 1451 bp; Supplementary Table S1). We searched for the best-fit sequence evolutionary model for each gene fragment using PartitionFinder v.2.1.1 (Canberra, Australia) [34]. We set the model selection to the Bayesian Information Criterion (BIC) and greedy search settings, and we standardized the estimation parameters of the partition model to unlinked branch length and fit to “Mr. Bayes” mode.

Here, the software recovered seven partitions based on a single non-coding and three coding codon’s positions (best sequence substitution models in Table 2). Using the information of best substitution models for the gene fragments, we then reconstructed the BI trees based on the four datasets using Mr Bayes v.3.2.7 (Rochester, NY, USA) [35]. For each dataset, we ran the analysis for 10 million iterations of MCMC, with four independent chains, and resampled at each 1000 generation of the run until the trees reached convergences. We defined convergence as split frequency values lower than 0.05 at the end of all analyses and values higher than 200 for the Estimated Sample Size (ESS) for each parameter in the diagnostic reports provided by the software.

Next, we constructed a haplotype network to identify the inheritance pattern between the sequences of our candidate species and its homologous sequences. Due to the strong posterior probability for the candidate species in the *COI* gene tree, and recommendations from the literature [36], we used the same dataset to analyze its haplotype distribution, consisting of 84 Hynobiids individuals (*n* taxa = 18) inferred from the *COI* gene fragment from individuals distributed across continental East Asia. Furthermore, we selected the *COI* alignment dataset due to its adequacy in the number of taxa for robust analysis in comparison to the concatenated dataset. We assigned the sequences to their respective species group and generated the haplotypes using DNAsp v.6.12.03 (Barcelona, Spain) [37]. We then determined the network of haplotypes using a statistical analysis of parsimony with TCS [38], implemented in PopART v.1.7 (Dunedin, New Zealand) [39]. Lastly, we mapped and visualized the distribution of the species involved in the haplotype network using QGIS v.2.18.0 (Chicago, IL, USA) [40].

Finally, to estimate the evolutionary divergence over sequence pairs between species, we analyzed 29 sequences of concatenated 16S rRNA, Cyt*b*, and *COI* using MEGA v.11.0.13 (State College, PA, USA) [41]. We assigned the sequence data to 18 groups based on species identity, and we removed all ambiguous positions for each sequence pair using the pairwise deletion option. There were a total of 1451 positions in the final dataset, and we computed the evolutionary divergence using the Maximum Composite Likelihood model [42].

### 2.5. Morphometry

For the morphological analyses, we measured the characters listed by Shen et al. [24], Lai and Lue [21], Borzée and Min [17], and Chen et al. [43] as they provide the largest dataset available for continental East Asian *Hynobius* species. See Figure 1 in Chen et al. [43] for a graphic representation of the following variables. We used digital calipers (model 1108–150, Insize; Suzhou, China) to the nearest 0.1 mm three times per individual and averaged, following the recommendations of Borzée and Min [17]. The measurements were: total length from the snout to the tail end (TOL); length of body from tip of snout to anterior angle of vent (SVL); tail length from anterior angle of vent to tip of tail (TL); tail height as the height of the tail at its highest point (TH); tail width as the width of the tail at its widest point (TW); head length from tip of snout to gular fold (HL); head width immediately posterior to jaw articulation (HW); snout length from the anterior border of the eye to the tip of the snout (SL); interocular distance, measured between medial margins of eyelids (IOD); laterally measured diameters of eyes (DE); head height as the height of the head at its highest point (HH); internarial distance, measured between the medial margins of the nares (IND); body width at axilla (BW); trunk length from axilla to groin (AG); forelimb length from anterior insertion to tip of second toe (FOL); hindlimb length from anterior insertion to tip of third toe (HIL). We also counted the number of costal grooves (COS) for all individuals, and the number of horny vomerine teeth (VT) for four individuals.

Ideally, a species description should include a morphological comparison between the new species and the most closely related one. Here, it would be a two-by-two comparison between the species described in this paper and *H. amjiensis* (see phylogenetic results). However, due to (1) the rarity of *H. amjiensis*, for instance, Chen et al. [44] found 16 individuals over eight years of surveys, (2) the urgency to describe the species to be able to protect it, (3) the robustness of the phylogenetic analyses (see phylogenetic results), and (4) the presence of great morphological variations; we provide a morphological description for the two adult individuals found, and a morphological key enabling the identification of the species. We do note the presence of morphological data in the literature for *H. maoershanensis* [43] and two Taiwanese species [21]; however, a two-by-two comparison with these species only would not be clarifying the taxonomy as these species are not phylogenetically or geographically close.

The species within the *H. chinensis* species complex, and ranging on the Chinese mainland, are *H. amjiensis*, *H. guabangshanensis*, *H. chinensis*, *H. maoershanensis*, and *H. yiwuensis*. We did not include individuals from northeast China, Taiwan Island, or the Japanese archipelago in the morphological comparisons as they were not closely related in the phylogenetic analyses. We then created a dichotomic key to enable the morphological identification of the species and the determination of discrete characters.

In addition, with the development of “technoecology” [45], 3D printing is becoming widely used, and 3D printed replicas can also benefit taxonomy and systematics [46]. Here, we provide a printable 3D model that can be downloaded online. We photographed an adult individual using a high-resolution camera (Nikon Z9 with a Nikkor MC 50 mm f 2.8 lens) in the wild but on a hard, permeable, and smooth substrate. We took multiple photographs from different angles, with a focus on capturing the characteristics and features of the species. The photos were imported into Autodesk Maya 2019 (Autodesk; San Francisco, CA, USA) as image planes, which were then used as reference images to ensure accuracy and consistency in the details of the model. Using the reference images as a guide, we created a base mesh using a polygon modeling technique. The base mesh was adjusted until it matched the overall shape of the salamander. The sculpting tools were then used to accurately refine and detail the features of the species. We then created a UV map and applied textures to the model, using photos of the specimen’s skin as a reference to create a realistic representation. The skeleton of the model was created by using rigging tools. The mesh was then skinned to the skeleton, allowing for a realistic pose of the model. The completed 3D model was exported from Maya in an OBJ format.

### 2.6. Egg and Larval Development

To ascertain that the development of the species does not deviate from that of the *H. chinensis* species complex, we documented the development of the species before the eggs hatched and after hatching at 16, 69, 74, 77, and 87 days. We aimed to document development between stages 29 and 37 when embryos are in the egg sac; tadpoles between stages 40 and 49 when free swimming with balancers; between stages 50 and 59, when the larvae are not yet using their developing hind legs and once after stage 60 when the larvae use their hind legs [47]. The salamanders were kept at 26 °C, in water boiled, and aged for at least 24 h in open-top buckets. Photographs were taken with a Z9 camera (Nikon, Tokyo, Japan) and a Z MC 50 mm f 2.8 lens (Nikkor, Nikon). 

### 2.7. Acoustic Signal

When probed, the individual captured in January sometimes emitted a vocalization. We managed to record the individual only once with a linear PCM recorder (Tascam DR-40; Santa Fe Springs, CA, USA) using the built-in microphone. The call was recorded at a sampling rate of 44.1 kHz with a 16-bit resolution. We analyzed the call properties following the method in Prasad et al. [48] using Raven Pro bioacoustics v.1.5 (Center for conservation bioacoustics; Ithaca, NY, USA).

## 3. Results

### 3.1. Molecular Analyses

The rates of evolutionary divergences between sequence pairs provided support for the significant variation between the candidate species and all 18 other species. The highest variation in the average base substitutions per site for sequence pairs was between the candidate species and *Hynobius amjiensis* (average rate = 0.098; marked with * in Table 3). The average divergence rate was comparable to eight other sequence pairs (bolded values in Table 3). Most importantly, the average divergence rate was comparatively higher than the rates of base substitution for the four following pairs: (i) *H. arisanensis* vs. *H. formosanus*, mean = 0.008; (ii) *H. maoershanensis* vs. *H. guabangshanensis*, mean = 0.021; (iii) *H. chinensis* vs. *H. guabangshanensis*, mean = 0.032; and (iv) *H. maoershanensis* vs. *H. chinensis* (mean: 0.035; Table 3).

**Figure 1:**
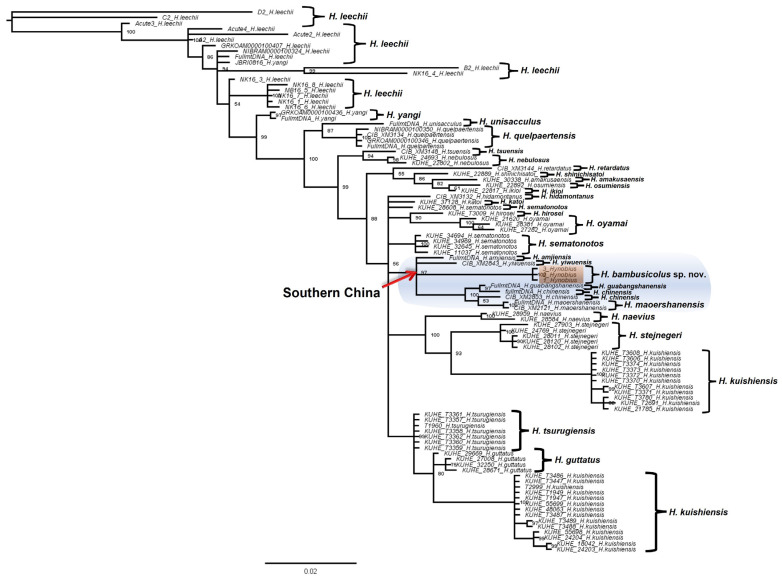
Bayesian Inference tree based on a 528 bp-long 16S rRNA fragment for 97 Hynobiidae salamanders in East Asia. Our results recovered *Hynobius bambusicolus* sp. nov. (orange shade) as monophyletic and sharing a sister relationship with the congeneric Hynobius species distributed in southern China (blue shade).

**Figure 2:**
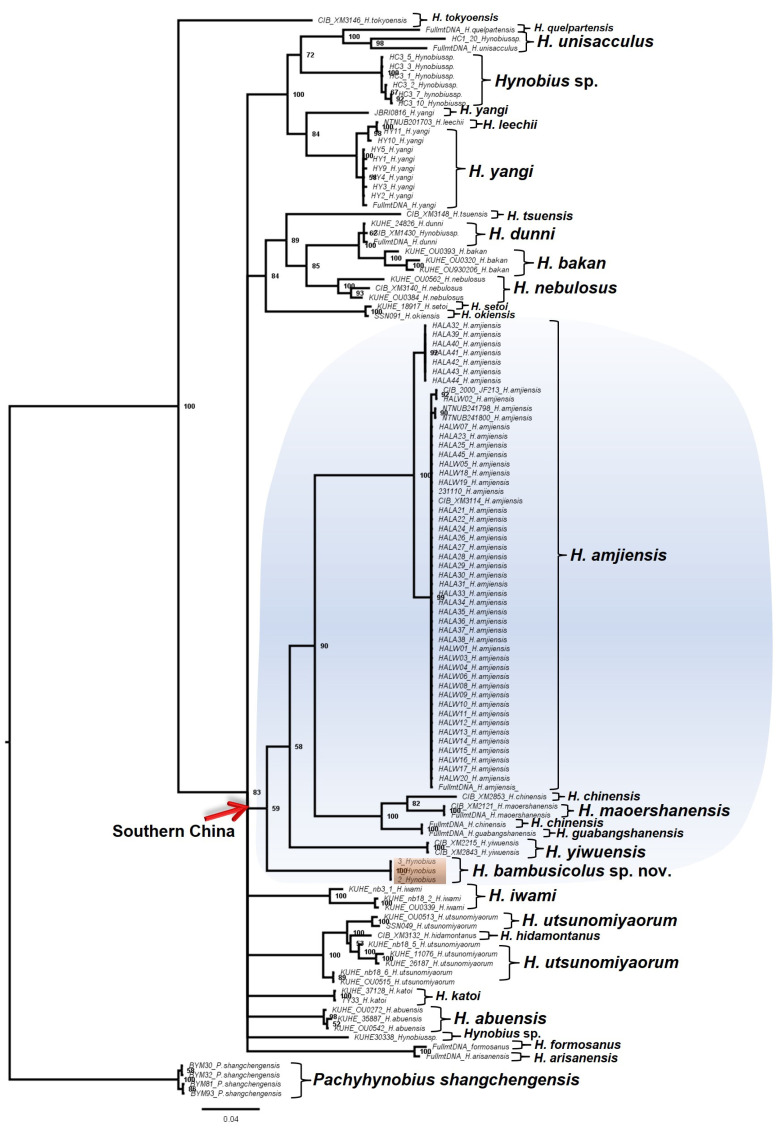
Bayesian Inference tree reconstructed from a 630 bp-long Cyt*b* gene fragment for 117 Hynobid salamanders distributed across East Asia. Our results recovered *Hynobius bambusicolus* sp. nov. (orange shade) as monophyletic and basal to the congeneric clades of *Hynobius* species distributed in southern China (blue shade).

**Figure 3:**
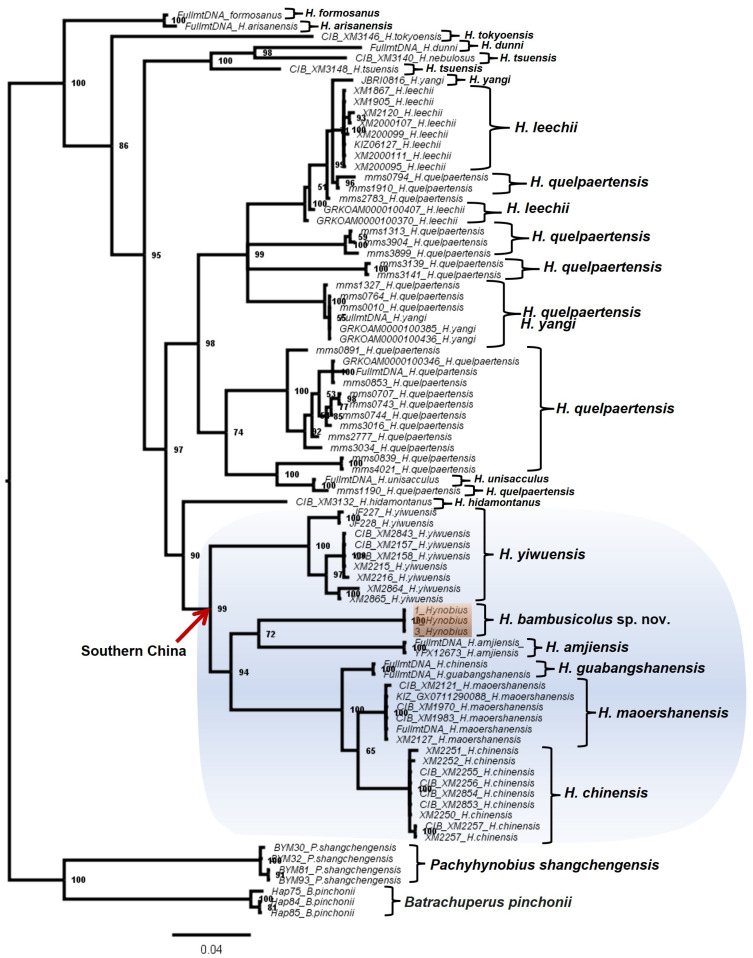
Bayesian Inference tree based on a 567 bp-long *COI* gene fragment for 84 Hynobid salamanders distributed across East Asia. Our results recovered *Hynobius bambusicolus* sp. nov. (orange shade) as monophyletic within the southern China group (blue shade) and sharing a sister relationship with *Hynobius amjiensis*.

**Figure 4:**
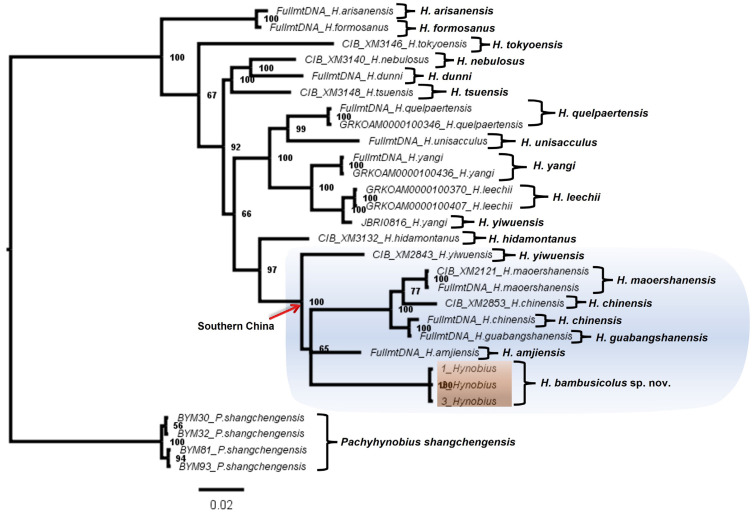
Bayesian Inference tree inferred from 1451 bp of 16S rRNA, Cyt*b,* and *COI* gene fragments of Hynobiid salamanders distributed across East Asia. Our analyses recovered *Hynobius bambusicolus* sp. nov. (orange shade) as clustered with the congeneric *Hynobius* distributed in southern China (blue shade).

Apart from the significant divergence in DNA sequences, our phylogeny recovered the candidate species as a deeply divergent clade amongst the East Asian Hynobiid (Figure 1, Figure 2, Figure 3 and Figure 4). All the BI trees, for single genes and concatenated fragments, coherently recovered the candidate species as monophyletic within the Southern Chinese group (posterior probability (PP): between 59% to 99%; Figure 1, Figure 2, Figure 3 and Figure 4). For the *COI* and concatenated trees, we recovered the candidate species as sister to *H. amjiensis* (PP: 97% and 65%; Figure 1).

**Figure 5:**
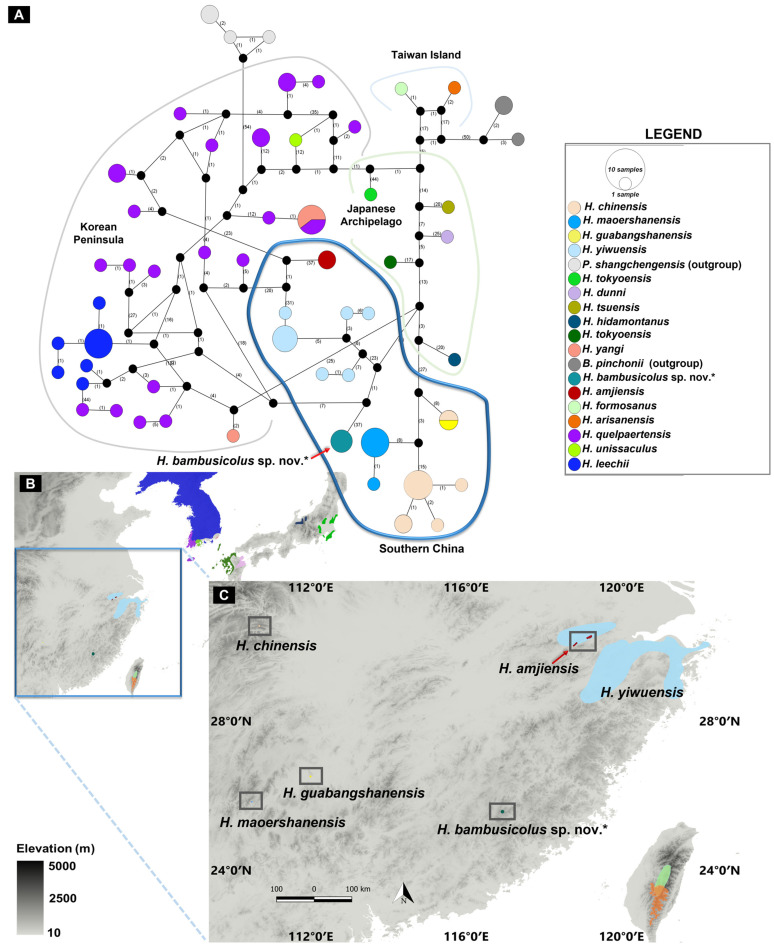
Haplotype network and distribution of Hynobiid salamanders distributed in East Asia. The haplotype analysis involved 84 Hynobiids individuals (*n* taxa = 18) inferred from the *COI* gene fragment (Figure 3). (**A**) TSC network and distribution of 55 haplotypes based on their geographic distribution. The number along each branch connecting haplotypes indicates the number of mutations. The asterisk (*) in the figure marks the focal *Hynobius* species described in this study, *Hynobius bambusicolus* sp. nov. (**B**) Distribution of all 18 *Hynobius* species in East Asia, matching with the haplotype network. The blue box highlights the southern Chinese clade of *Hynobius*. (**C**) Distribution of the southern Chinese clade of *Hynobius* species in which *Hynobius bambusicolus* sp. nov. is clustered. The small grey boxes highlight the restricted ranges of four *Hynobius* species within the clade.

Based on the gene fragment for *COI* (*n* taxa = 84), we obtained 55 haplotypes (h) from 567 sites representing 16 species of Hynobiid salamanders distributed across East Asia, with a haplotype diversity (Hd) of 0.98 (Figure 5). The haplotype distribution further revealed a shared relationship between the haplotype group of the candidate species and the geographically related haplotypes of Southern Chinese *Hynobius*. The haplotype network and distribution also clarified the deep genetic and geographic isolation between the candidate species and the related *H. amjiensis* and *H. maoershanensis*.

### 3.2. Morphometric results

**Figure 6:**
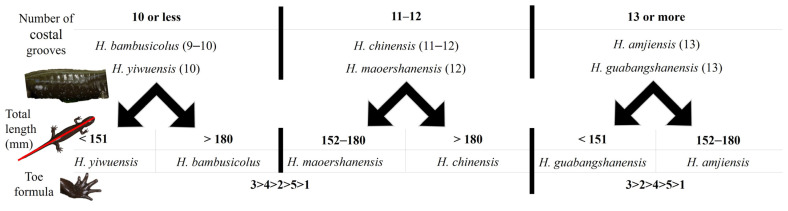
Species identification key for southern Chinese *Hynobius* species. With this morphological identification key, it is possible to identify adult *Hynobius* individuals from southern China, based on morphology and without invasive procedures. The data used are from AmphibiaChina.org and our study.

**Figure 7:**
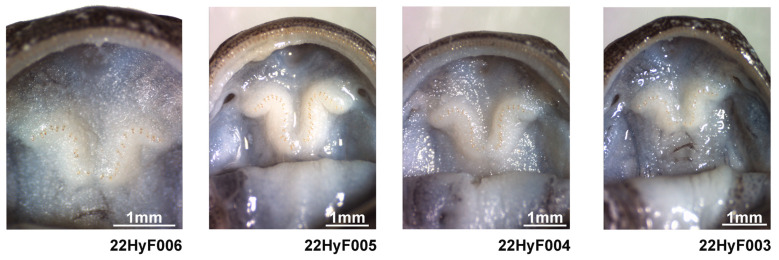
Vomeral teeth details for four juvenile *Hynobius bambusicolus* sp. nov. preserved in 70% alcohol. Vouchers were collected in Quxi village, Liancheng county, People’s Republic of China (25.566° N, 116.938° E).

The measurements of the adults highlighted the large size of the species (Table 4), surpassed only by *H. chinensis*, but a low number of coastal grooves, similar to *H. yiwuensis*. Finally, the toe formula was similar to that of three other species in the area (Figure 6). We found a variable number of vomeral teeth (four individuals), likely due to the partial development of the voucher specimen. The holotype was characterized by 12 pairs of vomeral teeth on the right and 13 on the left, the largest individual by 18 and 19 pairs, and the two other individuals by 17 and 16, and 12 and 13 pairs (Figure 7). As this character does not seem fixed yet in our voucher specimen, we do not use it for species identification. For the morphological identification key based on non-invasive identification of the species, we determined the number of costal grooves to be the first character of importance, enabling the assignment of the individual examined into either one of three categories: 10 or fewer grooves, 11 or 12 grooves, or 13 or more grooves (Figure 6). Once assigned to one of these categories, we could develop the following identification key:

- The combination of 10 or fewer grooves with the following:
  - A total length < 151 mm assigns the individual to *H. yiwuensis*;
  - A total length > 180 mm assigns the individual to *H. bambusicolus* sp. nov;
- The combination of 11 or 12 grooves with the following:
  - A total length < 180 mm assigns the individual to *H. maoershanensis*;
  - A total length > 180 mm assigns the individual to *H. chinensis*;
- The combination of 13 or more grooves with the following:
  - A total length < 151 mm assigns the individual to *H. guabangshanensis*;
  - A total length > 152 mm assigns the individual to *H. amjiensis*.

### 3.3. Species Description

Following the line of evidence based on molecular analyses for species-level divergence with other southern Chinese *Hynobius* species and a clearly different morphology evidenced by discrete characters, we formally describe the new species. Measurements are summarized in Table 4.

#### Nomenclature History

Among the species of *Hynobius* currently recognized [49], *Hynobius turkestanicus* Nikolskii is the only enigmatic taxon [50], and it is unlikely to be a member of the *Hynobius* genus [51]. Our focal taxa are also different from *Hynobius yunanicus*, which was invalidated [52] and later re-established [53], but as a synonym of *Pachyhynobius shangchengensis* [49]. The new species belongs to the group of lentic habitat breeders distributed in China and the Korean Peninsula, and morphological comparisons with the closest related species, *H. amjiensis*, and the geographically related species are presented in Figure 5.

*Hynobius bambusicolus* sp. nov. Wang, Othman, Qiu and Borzée

Synonymy:

“*Hynobius chinensis* (partim): [27]” pp. 218–229.

- Holotype

**Figure 8:**
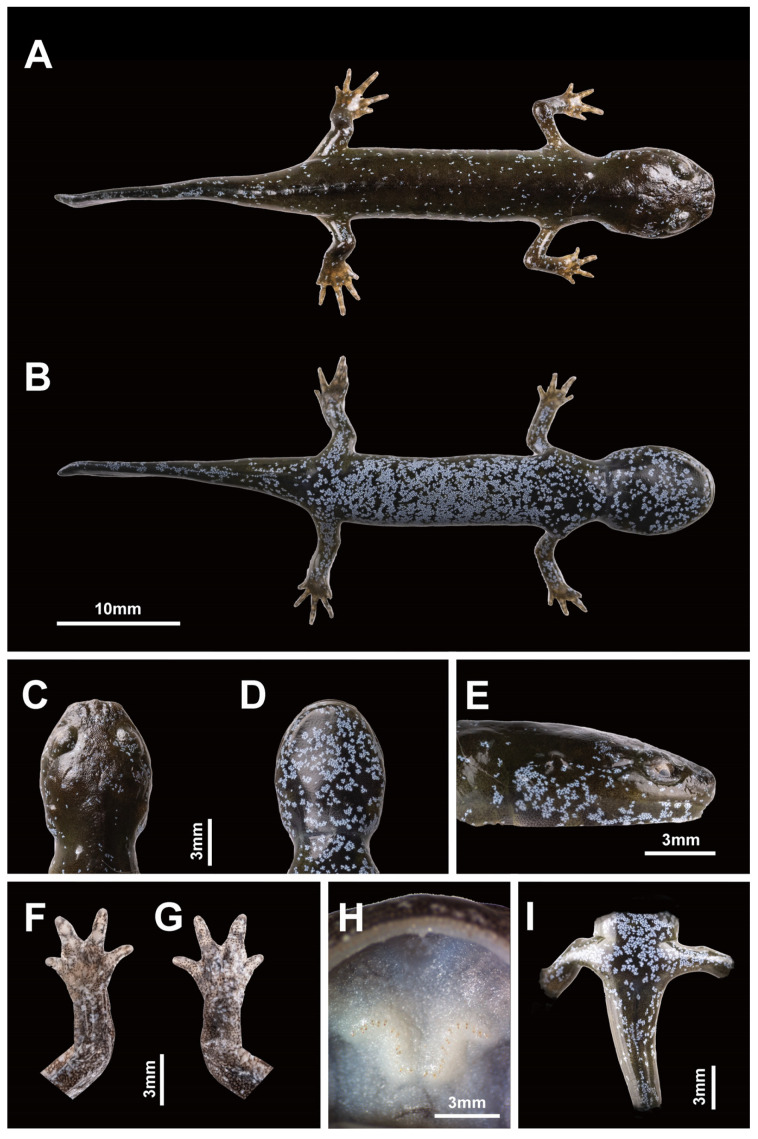
Holotype of *Hynobius bambusicolus* sp. nov. preserved in 70% alcohol. Voucher 22HyF006 collected in Quxi village, Liancheng county, People’s Republic of China (25.566° N, 116.938° E). Measurements and counts in Table 4. (**A**) Dorsal view. (**B**) Ventral view. (**C**) Head in dorsal view. (**D**) Head in ventral view. (**E**) Head in lateral view. (**F**) Opisthenar view of left hand. (**G**) Volar view of left hand. (**H**) Palatal region showing the vomerine tooth series (pale orange vertical structures on the white background at the center of the palate). (**I**) Ventral view of cloacal area.

Voucher 22HyF006; sub-adult collected by Zhenqi Wang and Zhixin Qiu on 26 January 2022 in Quxi village, Liancheng county, People’s Republic of China (25.566° N, 116.938° E; Figure 5). Measurements and counts are in Table 4 and Figure 8.

- Paratypes

**Figure 9:**
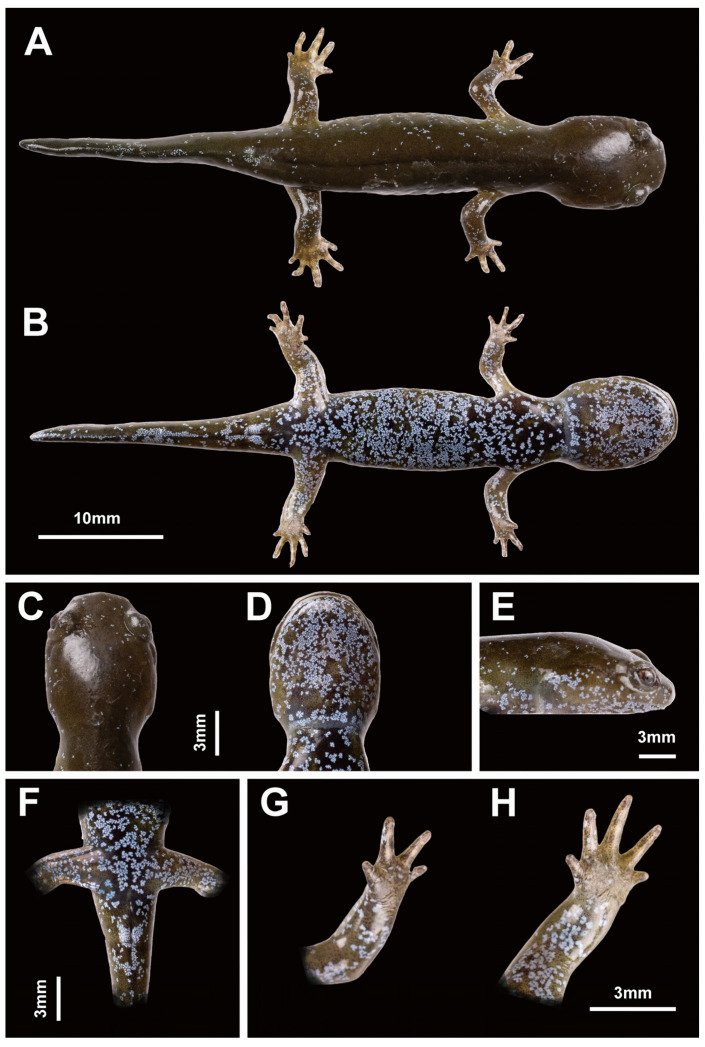
Paratype of *Hynobius bambusicolus* sp. nov. preserved in 70% alcohol. Voucher 22HyF003 collected in Quxi village, Liancheng county, People’s Republic of China (25.5661° N, 116.9386° E; Figure 5). Measurements and counts in Table 4. (**A**) Dorsal view. (**B**) ventral view. (**C**) Head dorsal view. (**D**) Head ventral view. (**E**) Head lateral view. (**F**) Ventral view of cloacal area. (**G**) Opisthenar view of left hand. (**H**) Volar view of left hand.

Vouchers 22HyF003, 22HyF004, 22HyF005. Sub-adults collected by Zhenqi Wang and Zhixin Qiu on 26 January 2022 in Quxi village, Liancheng county, People’s Republic of China (25.566° N, 116.938° E; Figure 5). Measurements and counts are in Table 4 and Figure 9.

- Etymology

The species was first found in Quxi village, Liancheng county, in the west of Fujian province in China. The name *H*. *bambusicolus* sp. nov. comes from the habitat of the holotype and the only known habitat type for the species: bamboo forests. The vernacular name of the species, Fujian Bamboo Salamander, reflects the scientific name of the species, as does its Chinese name: 虚竹小鲵 (pronounced: Xū Zhú Xiǎo Ní). This salamander is named after a main character from Jin Yong’s swordsman fiction “The semi gods and semi devils” [54], with Xuzhu (虚竹) as the main character and where “虚” [xū] means humble, and “竹” [zhú] means bamboo. This character, Xuzhu, was an unknown Shaolin monk, but he inherited the powers of the leader of the Carefree by coincidence and started its legendary journey.

- Identity, diagnosis, and distribution

**Figure 10:**
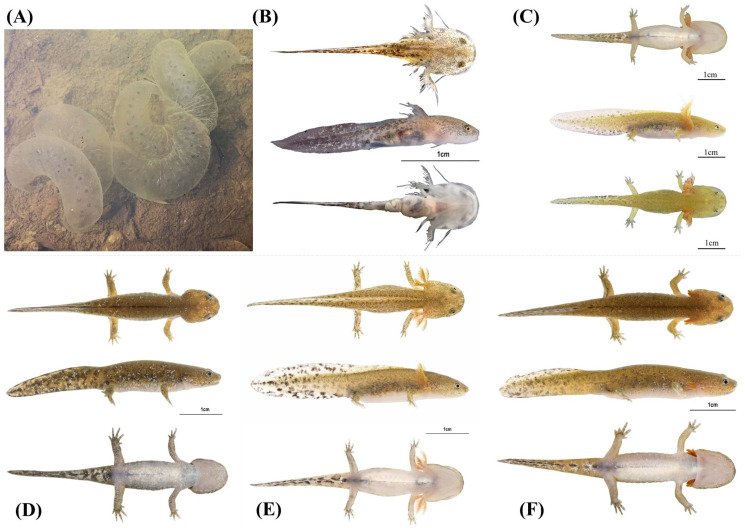
Representative developmental stages for eggs and larvae of *Hynobius bambusicolus* sp. nov. from Fujian, China. (**A**) pre-hatching; (**B**) 16 days old. (**C**) 69 days old. (**D**) 87 days old. (**E**) 74 days old. (**F**) 77 days old.

**Figure 11:**
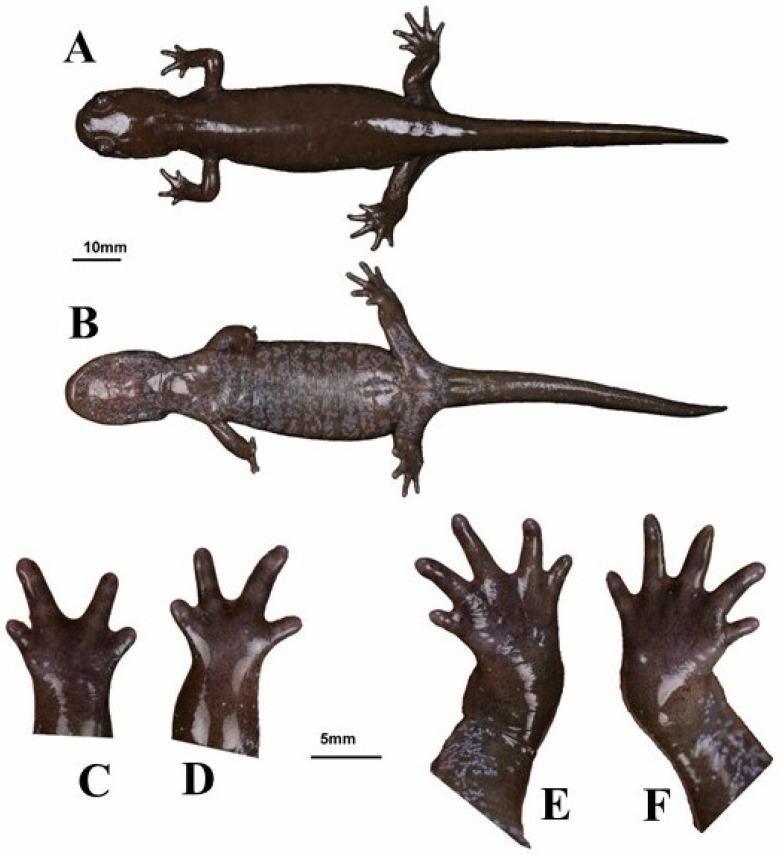
Example of *Hynobius bambusicolus* sp. nov. adult in life. Voucher 22HyF001 from Quxi village, Liancheng county, People’s Republic of China (25.5661° N, 116.9386° E; Figure 5). Measurements and counts in Table 4. (**A**) Dorsal view. (**B**) ventral view. (**C**) Opisthenar view of left hand. (**D**) Opisthenar view of right hand. (**E**) Opisthenar view of left foot. (**F**) Opisthenar view of right foot.

**Figure A1:**
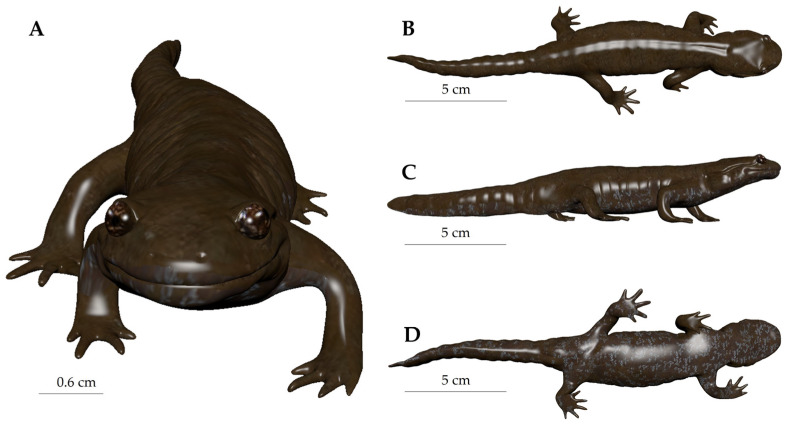
Representation of the 3D model. (**A**) Frontal view. (**B**) Dorsal view. (**C**) lateral view. (**D**) Ventral view.

To date, the species is known from its type locality only, Quxi village, Liancheng county (Figure 5). Larvae are typical of *Hynobius* larvae in shape and color and do not differ from other *Hynobius* species in the region in their development (Figure 10). The embryos develop in egg sacs (Figure 10A), larvae first swimming freely with balancers (Figure 10B; shown at day 16), then develop non-functional hind limbs (Figure 10C; shown at day 69), which slowly become functional (Figure 10D,E; shown at day 74 and 77), and the gills regress before metamorphosis (Figure 10F; shown at day 84). Juveniles are brown, darkening with age, with a large variation in blue speckles on their dorsum, which disappears as they age (Figure 9). Adults of the species are uniform dark chocolate, with light grey and bluish speckles on the venter (Figure 11). Identification is best assessed based on location, although discrete morphological characters include the combination of 10 or fewer costal grooves with a total length > 180 mm (Figure 6). To facilitate the identification and further study, the OBJ file of this model can be downloaded (Supplementary File S1). The visual representation of the model is provided in Appendix A (Figure A1).

- ZooBank registration

We hereby state that the present paper has been registered to the Official Register of Zoological Nomenclature (ZooBank) under LSID: urn:lsid:zoobank.org:pub: 9047D736-3394-4B36-AE97-CEEB3359B36D. The new species name *Hynobius bambusicolus* sp. nov., has been registered under LSID: urn:lsid:zoobank.org:act:18CAEC61-DF60-4401-91C5-FEF5614FA08C.

- Nomenclatural acts

The electronic edition of this article conforms to the requirements of the amended

International Code of Zoological Nomenclature, and hence the new names contained herein are available under that code from the electronic edition of this article. This published work and the nomenclatural act it contains have been registered in ZooBank, the online registration system for the ICZN. The ZooBank LSIDs (Life Science Identifiers) can be resolved, and the associated information viewed through any standard web browser by appending the LSID to the prefix “http://zoobank.org/”. The LSID for this publication is urn:lsid:zoobank.org:pub:9047D736-3394-4B36-AE97-CEEB3359B36D. The electronic edition of this work was published in a journal with an ISSN and has been archived and is available from the following digital repositories: PubMed Central.

- Habitat and behavior

**Figure 12:**
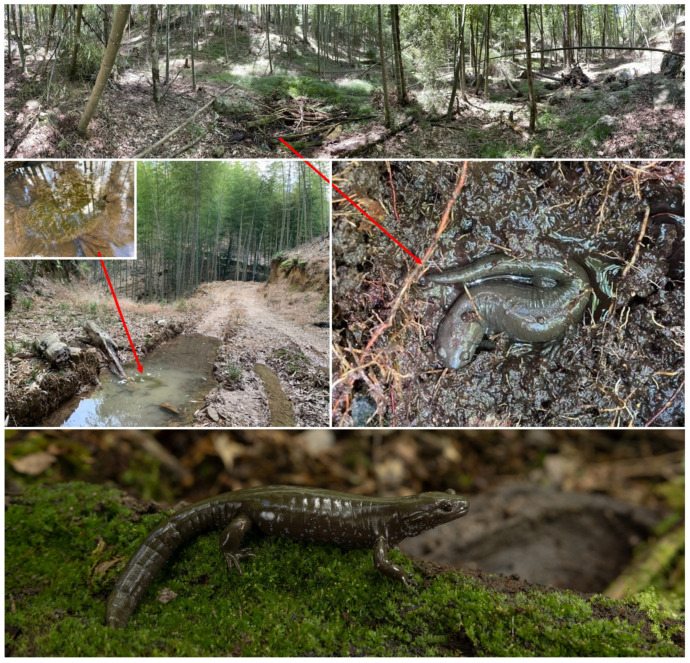
Natural habitat and oviposition site for *Hynobius bambusicolus* sp. nov. The site is in Quxi village, Liancheng county, People’s Republic of China (25.5661° N, 116.9386° E).

**Figure 13:**
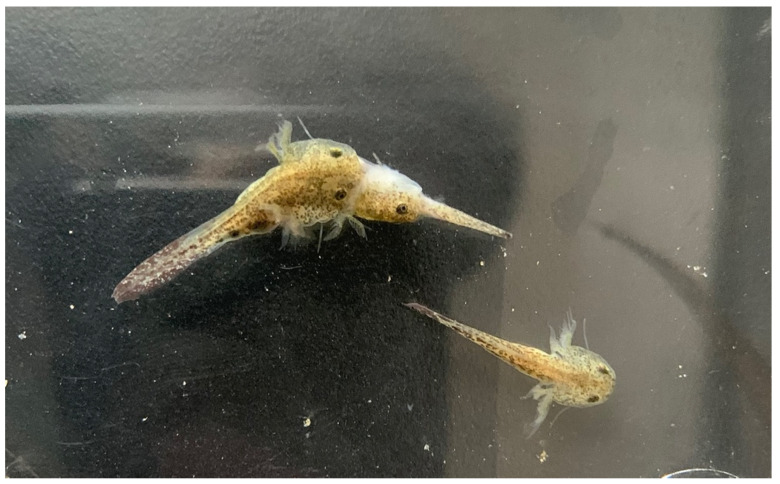
Example of cannibalism in *Hynobius bambusicolus* sp. nov. larvae.

*Hynobius bambusicolus* breeds in shallow pools in bamboo forests (Figure 12) above 1400 m above sea level. All eggs were observed in ruts made from tire tracks. The puddles were 5 to 12 cm deep, and most egg sacs were laid about 10 cm deep without being attached to the substrate, containing between 21 and 27 eggs each. Adult salamanders were found under logs, stones, and dead leaves, in wet soil and humus. These shelters were surrounded by weeds and dry branches in waterlogged areas, and we did not find any individuals on the surface, even at night (Figure 12). Larvae were fed with bloodworms, but some individuals still cannibalized their kin (Figure 13). Once metamorphosed, recently metamorphosed individuals raised their tails when startled, a behavior to divert predation from vital organs [55].

**Figure 14:**
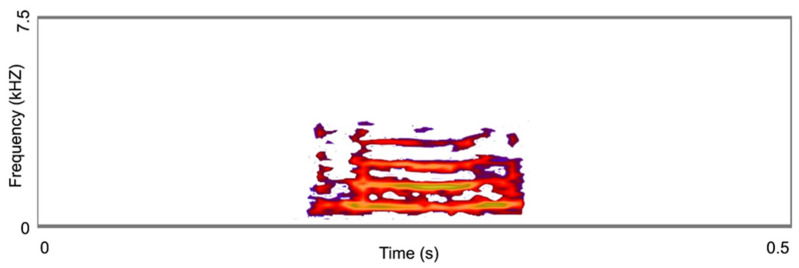
Spectrogram of the acoustic signal release by *Hynobius bambusicolus* sp. nov. Dense yellow-orange colour displays the harmonic-like pattern of the acoustic signal. Schematic illustrations based on the data extracted with FFT size = 1024 pts. (Hanning window, 43.1 Hz resolution).

The individual recorded produced a single type of call, with a very short duration (153.40 ms) and of low frequency (peak frequency of 129.20 Hz). The vocalization was composed of four strong harmonics (Figure 14), with a peak amplitude of 403 U and a maximum entropy of 3.473 bits. While highly unusual, underwater vocalizations have been reported in salamanders, such as *Siren intermedia* [56]. In this case, the vocalizations were produced by a male being probed, suggesting it could be submissive or alarm calls. In our recording, the individual emitted a similar “alarm call”/“squeak” while half submerged in the water, maybe as an agonistic signal.

## 4. Discussion and Conclusions

Here we described a Hynobiid salamander species, *Hynobius bambusicolus,* the Fujian Bamboo Salamander, based on deeply divergent molecular analyses, non-overlapping morphological characters, and vastly segregated distribution. Field identification of *Hynobius* individuals based on morphology is not always easy [17], but discrete morphological characters are present to identify *H. bambusicolus* (Figure 6). The easiest character to identify the species perhaps remains the geographic location as the range of the species does not overlap with that of the other *Hynobius* species, with a gap of several hundred kilometers (Figure 5).

Our results, through the phylogenetic trees, haplotype network, and comparative pairwise genetic distance, show that *H. bambusicolus* is alternatively the most divergent and earliest-branching species among Southern Chinese *Hynobius* clades (Cyt*b* tree; Figure 2), nested with the other Southern Chinese *Hynobius* clades (16S rRNA and concatenated mtDNA trees; Figure 1 and Figure 4) and only the sister taxon to *H. amjiensis* (*COI* tree; Figure 3). The stem emergence of Southern Chinese *Hynobius* clades (*H. amjiensis* and *H. chinensis*) is dated to c. 20 mya [57], and *H. bambusicolus* is, therefore, an ancient lineage, likely to have seen its distribution regularly shift, expand, and contract with paleogeographic and climatic variations [58]. The area is inhabited by other Caudata, and competition between *H. bambusicolus* and other genera, such as *Pachytriton* and *Paramesotriton*, is not impossible.

The restricted distribution of *H. bambusicolus* is also a negative point to the survival of the species. The species is micro-endemic, presumably composed of a single known relic population with an apparently incredibly small population size, similar to other south Chinese *Hynobius* species (e.g., [44]). The genus is likely to have distributed south broadly since the early Miocene [59], and withdrawn to higher elevations, similarly to other amphibians shifting distribution [60], due to climatic variations as most representatives of the genus are cold-adapted, and the mid-Miocene vegetation in Fujian was tropical [61]. For instance, specific genes in *H. chinensis* are upregulated as a response to cold temperatures [62]. *Hynobius bambusicolus*, however, is adapted to sub-tropical bamboo forests, although cold preference or tolerance for spawning is still a trait in the species, as two egg masses were found in January 2023 at the same site on a snowy day. 

The species is known from a single locality, and surveys in 2023 of all water bodies in the area where the species could potentially spawn did not result in more than five extremely small water bodies. As there were eight egg sacks at the known site (therefore four females), even considering this number as a constant for each water body, it would be a maximum of 20 breeding females, and therefore a population size likely to be well below 200 breeding individuals, matching with the criteria B1, B2, and C2 for Critically Endangered following the recommendation of the IUCN Red List of Threatened Species [63]. Due to the threatened status of the species, establishing an ex situ population could help prevent the extinction of the species in the face of growing climate instability and stochastic extinction risks.

Due to the rarity of this new species, we urge all hobbyists to avoid collecting this salamander or divulging local information and resisting any trade. The breeding site is within a planted bamboo forest, surrounded by mixed forest, and the distribution of the species is likely to have been impacted by bamboo plantation and harvest over the last decades or centuries. A site where the species was found about 10 years ago by a ranger on the opposite side of the valley is now entirely dry and unlikely to support a breeding population, making habitat loss the main threat to the species. Climate change is also most likely to impact the species, as seen in other Hynobiids [64], and we can expect the distribution of the species to be contracting. It is, therefore, important to lower other stresses as amphibians can cope with a low number of conservation pressures, but populations crash when there are too many stress factors [65]. As the species is occurring in bamboo forests exploited by humans, we recommend reducing the use of herbicides and restricting water pumping in the area. In addition, restoring the habitat through the creation of additional artificial shallow ponds or rehabilitating old water reservoirs found throughout the bamboo plantation will help boost population growth [66].

## Figures and Tables

**Table 1:** PCR information and protocols to amplify DNA fragments. Three mtDNA fragments were selected to study the phylogenetic position of the candidate *Hynobius* species. The primer pair for the Cyt*b* fragment was designed in-house, and the other sequences come from the literature. The length of each PCR amplicon is estimated in base pairs.

Fragment	Primer	5′-3′	Length	Source	PCR Condition
mtDNA—*COI*	LCO1490	GGTCAACAAATCATAAAGATATTG	680 bp	[31]	95 °C for 3 min, followed by 35 cycles of 95 °C for 30 s, 53 °C for 30 s and 72 °C for 40 s, and final elongation at 72 °C for 7 min
	HCO2198	TAAACTTCAGGGTGACCAAAAAATCA	680 bp	[31]	
16S rRNA	L02510	CGCCTGTTTATCAAAAACAT	500 bp	[32]	95 °C for 3 min, followed by 35 cycles of 95 °C for 30 s, 53 °C for 30 s and 72 °C for 40 s, and final elongation at 72 °C for 7 min
	H03063	CTCCGGTTTGAACTCAGATC	500 bp	[33]	
mtDNA-Cyt*b* (in-house design)	CytbHy-F1	TGTAGACCTCCCAACCCCC	780 bp	This study	95 °C for 5 min, followed by 35 cycles of 94 °C for 30 s, 55–60 °C for 30 s and 72 °C for 60 s, and final elongation at 72 °C for 10 min
	CytbHy-R1	CGTAGGCGAATAAGAAATACCACT	780 bp	This study	

**Table 2:** Sequence substitution model. Best sequence substitution model for each gene fragment of the candidate *Hynobius* species are related sequences used for the reconstruction of the Bayesian Inference trees using Mr. Bayes v.3.2.7 [35], predicted and configured in PartitionFinder v. 2.1.1 [34].

Mitochondrial Gene Fragment	Type	Partition’s Strategy	Best Sequence Substitution Model
16S rRNA	Non-coding ribosomal	1–528 bp	HKY + I + G
Cyt*b*	Protein-coding	Exon by three codons’ position (1–630 bp; 2–630 bp; 3–630 bp)	GTR + I + G
*COI*	Protein-coding	Exon by three codons’ position (1–567 bp; 2–567 bp; 3–567 bp)	GTR + I + G

**Table 3:** Matrix of best substitution rates for evolutionary divergence between the candidate *Hynobius* species and congeneric species. The congeneric *Hynobius* species used in this study are related Hynobiid salamanders distributed in East Asia. We inferred the genetic distance from mitochondrial 16S rRNA, Cyt*b,* and *COI* (1451 bp) from 29 taxa representing 18 species. The rate of base substitution provides support to the evolutionary divergence between sequences of *Hynobius bambusicolus* sp. nov. and all 17 species compared. The lowest genetic distance is between the focal species and *H. amjiensis* (0.098; marked with *), but it is higher than the value from four species-pair comparisons (marked with #). All genetic distance values lower than the mean of pairwise difference for *H. bambusicolus* sp. nov. (0.098) are in bold in the table.

Species		1	2	3	4	5	6	7	8	9	10	11	12	13	14	15	16	17	18
1	*H. bambusicolus* sp. nov.	N/A																	
2	*P. shangchengensis*	0.166																	
3	*H. maoershanensis*	0.113	0.170																
4	*H. yiwuensis*	0.102	0.157	0.106															
5	*H. chinensis*	0.106	0.166	**0.035 #**	0.096														
6	*H. hidamontanus*	0.101	0.146	**0.093**	**0.090**	0.091													
7	*H. nebulosus*	0.119	0.149	0.106	0.100	0.106	0.083												
8	*H. tokyoensis*	0.138	0.173	0.132	0.128	0.126	0.118	0.126											
9	*H. tsuensis*	0.115	0.147	0.106	0.096	0.101	0.086	**0.074**	0.126										
10	*H. amjiensis*	0.098 *	0.161	**0.092**	0.096	0.090	0.095	0.102	0.132	0.099									
11	*H. arisanensis*	0.118	0.145	0.121	0.110	0.121	0.109	0.104	0.121	0.098	0.122								
12	*H. dunni*	0.122	0.156	0.109	0.107	0.106	0.091	**0.060**	0.121	**0.077**	0.099	0.105							
13	*H. formosanus*	0.115	0.144	0.121	0.106	0.122	0.108	0.104	0.117	0.100	0.123	**0.008 #**	0.104						
14	*H. guabangshanensis*	0.106	0.161	**0.032 #**	**0.093**	**0.021 #**	**0.083**	0.103	0.127	0.098	0.091	0.117	0.106	0.118					
15	*H. quelpaertensis*	0.114	0.146	0.105	0.099	0.100	**0.085**	0.092	0.123	**0.088**	0.095	0.111	**0.094**	0.107	0.095				
16	*H. unisacculus*	0.116	0.141	0.105	0.096	0.102	0.095	0.093	0.120	0.092	0.096	0.113	**0.096**	0.110	0.102	**0.062**			
17	*H. yangi*	0.104	0.147	0.114	0.101	0.107	0.083	**0.088**	0.122	**0.088**	0.099	0.105	**0.096**	0.104	0.108	**0.075**	**0.073**		
18	*H. leechii*	0.100	0.148	0.107	0.099	0.104	0.081	0.089	0.121	0.089	0.098	0.105	0.097	0.104	0.107	0.066	0.070	0.036	N/A

**Table 4:** Morphological measurements. Morphological measurements for two adult and four juvenile *Hynobius bambusicolus* sp. nov. from Fujian, China. The measurements were taken three times per individual and averaged for consistency.

Age	Adults		Juveniles			
Type	Wild	Wild	Paratype	Paratype	Paratype	Holotype
ID	22HyF001	22HyF002	22HyF003	22HyF004	22HyF005	22HyF006
TOL	191.57	196.94	48.06	48.38	50.43	55.11
SVL	136.36	137.81	28.39	27.72	31.32	34.05
TL	55.21	59.13	19.57	18.74	20.32	21.73
HL	22.78	21.86	7.27	8.48	8.25	10.32
HW	18.57	15.84	6.22	6.80	6.82	7.82
IOD	6.19	5.25	2.67	2.92	2.83	3.44
IND	6.29	5.77	3.16	3.29	2.72	3.16
BW	17.75	16.08	5.24	6.24	6.83	7.09
AG	52.07	51.06	16.46	16.14	17.95	21.04
FOL	21.13	20.77	6.49	6.73	5.89	8.08
HIL	25.53	23.08	6.38	6.30	8.73	7.77
COS	9	10	9	9	10	9
SL	19.78	20.82	4.72	5.47	4.44	5.37
TH	10.12	10.52	3.26	4.02	3.06	3.49
TW	11.03	10.90	2.21	2.29	2.83	2.48
HH	10.50	10.99	3.81	3.91	3.92	3.70
DE	5.31	4.64	2.44	2.31	2.80	2.54

## Data Availability

The morphological data are in Table 4, and the genetic sequences are in Supplementary Table S1. Accession numbers and references for all DNA sequences used for the bioinformatic analyses. DNA sequences of the candidate *Hynobius* species generated in this study and homologous sequences are retrieved from Genbank.

## Supplementary Materials

The following supporting information can be downloaded at: https://www.mdpi.com/article/10.3390/ani13101661/s1, Table S1: GenBank accession numbers; File S1: obj 3D model.

## References

[1] Wake D.B., Vredenburg V.T. (2008). Are we in the midst of the sixth mass extinction? A view from the world of amphibians. Proc. Natl. Acad. Sci. USA.

[2] Ceballos G., Ehrlich P.R., Barnosky A.D., García A., Pringle R.M., Palmer T.M. (2015). Accelerated modern human–induced species losses: Entering the sixth mass extinction. Sci. Adv..

[3] Smith F.A., Elliott Smith R.E., Lyons S.K., Payne J.L. (2018). Body size downgrading of mammals over the late Quaternary. Science.

[4] Manne L.L., Pimm S.L. (2001). Beyond eight forms of rarity: Which species are threatened and which will be next?. Anim. Conserv..

[5] Régnier C., Achaz G., Lambert A., Cowie R.H., Bouchet P., Fontaine B. (2015). Mass extinction in poorly known taxa. Proc. Natl. Acad. Sci. USA.

[6] Tedesco P.A., Bigorne R., Bogan A.E., Giam X., Jézéquel C., Hugueny B. (2014). Estimating how many undescribed species have gone extinct. Conserv. Biol..

[7] Brodie J.F., Aslan C.E., Rogers H.S., Redford K.H., Maron J.L., Bronstein J.L., Groves C.R. (2014). Secondary extinctions of biodiversity. Trends Ecol. Evol..

[8] Rix M.G., Huey J.A., Main B.Y., Waldock J.M., Harrison S.E., Comer S., Austin A.D., Harvey M.S. (2017). Where have all the spiders gone? The decline of a poorly known invertebrate fauna in the agricultural and arid zones of southern Australia. Austral Entomol..

[9] Rinawati F., Stein K., Lindner A. (2013). Climate change impacts on biodiversity—The setting of a lingering global crisis. Diversity.

[10] Otto S.P. (2018). Adaptation, speciation and extinction in the Anthropocene. Proc. R. Soc. B.

[11] Wang Z., Zeng J., Meng W., Lohman D.J., Pierce N.E. (2021). Out of sight, out of mind: Public and research interest in insects is negatively correlated with their conservation status. Insect Conserv. Divers..

[12] Sodhi N.S., Brook B.W., Bradshaw C.J., Levin S.A., Carpenter S.R., Godfray H.C.J., Kinzig A.P., Loreau M., Losos J.B., Walker B., Wilcove D.S. (2009). Causes and consequences of species extinctions. The Princeton Guide to Ecology.

[13] Lindenmayer D.B., Fischer J. (2013). Habitat Fragmentation and Landscape Change: An Ecological and Conservation Synthesis.

[14] Wang K., Ren J., Chen H., Lyu Z., Guo X., Jiang K., Chen J., Li J., Guo P., Wang Y. (2020). The updated checklists of amphibians and reptiles of China. Biodivers. Sci..

[15] Moura M.R., Jetzt W. (2021). Shortfalls and opportunities in terrestrial vertebrate species discovery. Nat. Ecol. Evol..

[16] Button S., Borzée A. (2021). An integrative synthesis to global amphibian conservation priorities. Glob. Chang. Biol..

[17] Borzée A., Min M.-S. (2021). Disentangling the impact of speciation, sympatry and island effect on the morphology of seven *Hynobius* sp. salamanders. Animals.

[18] Matsui M., Misawa Y., Yoshikawa N., Nishikawa K. (2022). Taxonomic reappraisal of *Hynobius tokyoensis*, with description of a new species from northeastern Honshu, Japan (Amphibia: Caudata). Zootaxa.

[19] Sugawara H., Naito J.I., Iwata T., Nagano M. (2022). Molecular phylogenetic and morphological problems of the Aki Salamander *Hynobius akiensis*: Description of two new species from Chugoku, Japan. Bull. Kanagawa Prefect. Mus..

[20] Sugawara H., Fujitani T., Seguchi S., Sawahata T., Nagano M. (2022). Taxonomic re-examination of the Yamato Salamander *Hynobius vandenburghi*: Description of a new species from Central Honshu, Japan. Bull. Kanagawa Prefect. Mus..

[21] Lai J.S., Lue K.Y. (2008). Two new *Hynobius* (Caudata: Hynobiidae) salamanders from Taiwan. Herpetologica.

[22] Günther A.C.L.G. (1889). Third contribution to our knowledge of reptiles and fishes from the upper Yangtze-Kiang. Ann. Mag. Nat. Hist..

[23] Gu H.-q., Qian Y., Zhao E.-M., Zhao K.-t. (1992). A new species of *Hynobius*-*Hynobius amjiensis*. Animal Science Research: A Symposium Issued to Celebrate the 90th Birthday of the Professor Mangven Ly Chang.

[24] Shen Y.-h., Deng X.-j., Wang B. (2004). A new hynobiid species, *Hynobius guabangshanensis*, from Hunan Province, China (Amphiba: Hynobiidae). Acta Zool. Sin..

[25] Zhou F., Jiang A.-W., Jiang D.-b. (2006). A new species of the genus *Hynobius* from Guangxi Zhuang Autonomous Region, China (Caudata, Hynobiidae). Acta Zootaxonomica Sin..

[26] Cai C.-m. (1985). A survey of tailed amphibians for Zhejiang, with description of a new species of *Hynobius*. Acta Herpetol. Sin..

[27] Hu S., Fei L., Ye C. (1978). Investigation report of amphibians in Fujian. Research Material of Amphibians and Reptiles.

[28] Fei L., Hu S., Ye C., Huang Y. (2006). Fauna Sinica: Amphibia.

[29] IUCN (1989). IUCN Policy Statement on Research Involving Species at Risk of Extinction.

[30] Kusano T., Miyashita K. (1984). Dispersal of the salamander, *Hynobius nebulosus tokyoensis*. J. Herpetol..

[31] Folmer O., Black M., Hoeh W., Lutz R., Vrijenhoek R. (1994). DNA primers for amplification of mitochondrial cytochrome c oxidase subunit I from diverse metazoan invertebrates. Mol. Mar. Biol. Biotechnol..

[32] Palumbi S., Hillis D., Moritz C., Mable B. (1996). Nucleic acids II: The polymerase chain reaction. Molecular Systematics.

[33] Rassmann K. (1997). Evolutionary age of the Galapagos iguanas predates the age of the present Galapagos Islands. Mol. Phylogenet. Evol..

[34] Lanfear R., Frandsen P.B., Wright A.M., Senfeld T., Calcott B. (2017). PartitionFinder 2: New methods for selecting partitioned models of evolution for molecular and morphological phylogenetic analyses. Mol. Biol. Evol..

[35] Ronquist F., Teslenko M., van der Mark P., Ayres D., Darling A., Höhna S., Larget B., Liu L., Suchard M., Huelsenbeck J. (2012). MrBayes 3.2: Efficient Bayesian phylogenetic inference and model choice across a large model space. Syst. Biol..

[36] Xia Y., Gu H.F., Peng R., Chen Q., Zheng Y.C., Murphy R.W., Zeng X.M. (2012). COI is better than 16S rRNA for DNA barcoding Asiatic salamanders (Amphibia: Caudata: Hynobiidae). Mol. Ecol. Resour..

[37] Rozas J., Ferrer-Mata A., Sánchez-DelBarrio J., Guirao-Rico S., Librado P., Ramos-Onsins S., Sánchez-Gracia A. (2017). DnaSP 6: DNA sequence polymorphism analysis of large data sets. Mol. Biol. Evol..

[38] Templeton A.R., Crandall K.A., Sing C.F. (1992). A cladistic analysis of phenotypic associations with haplotypes inferred from restriction endonuclease mapping and DNA sequence data. III. Cladogram estimation. Genetics.

[39] Leigh J.W., Bryant D. (2015). POPART: Full-feature software for haplotype network construction. Methods Ecol. Evol..

[40] QGIS Development Team QGIS Geographic Information System.; Open Source Geospatial Foundation Project. http://qgis.osgeo.org.

[41] Tamura K., Stecher G., Kumar S. (2021). MEGA 11: Molecular Evolutionary Genetics Analysis Version 11. Mol. Biol. Evol..

[42] Tamura K., Nei M., Kumar S. (2004). Prospects for inferring very large phylogenies by using the neighbor-joining method. Proc. Natl. Acad. Sci. USA.

[43] Chen H., Bu R., Ning M., Yang B., Wu Z., Huang H. (2022). Sexual Dimorphism in the Chinese Endemic Species *Hynobius maoershanensis* (Urodela: Hynobiidae). Animals.

[44] Chen C., Yang J., Wu Y., Fan Z., Lu W., Chen S., Yu L. (2016). The breeding ecology of a critically endangered salamander, *Hynobius amjiensis* (Caudata: Hynobiidae), Endemic to Eastern China. Asian Herpetol. Res..

[45] Allan B.M., Nimmo D.G., Ierodiaconou D., Wal J.V.D., Koh L.P., Ritchie E.G. (2018). Futurecasting ecological research: The rise of technoecology. Ecosphere.

[46] Fernando M.d.F.L. (2018). 3D print so more scholars can access unique specimens. Nature.

[47] Iwasawa H., Yamashita K. (1991). Normal stages of development of a hynobiid salamander, *Hynobius nigrescens* Stejneger. Jpn. J. Herpetol..

[48] Prasad V.K., Chuang M.F., Das A., Ramesh K., Yi Y., Dinesh K.P., Borzée A. (2022). Coexisting good neighbours: Acoustic and calling microhabitat niche partitioning in two elusive syntopic species of balloon frogs, *Uperodon systoma* and *U. globulosus* (Anura: Microhylidae) and potential of individual vocal signatures. BMC Zool..

[49] Frost D.R. (2022). Amphibian Species of the World: An Online Reference.

[50] Kuzmin S.L., Dunayev E.A. (2000). On the problem of the type territory of the Turkestanian Salamander (*Hynobius turkestanicus* Nikolsky, 1909). Adv. Amphib. Res. Former Sov. Union.

[51] Min M.-S., Baek H., Song J.-Y., Chang M., Poyarkov Jr N. (2016). A new species of salamander of the genus *Hynobius* (Amphibia, Caudata, Hynobiidae) from South Korea. Zootaxa.

[52] Xiong J.L., Chen Q., Zeng X.M., Zhao E.M., Qing L.Y. (2007). Karyotypic, morphological, and molecular evidence for *Hynobius yunanicus* as a synonym of *Pachyhynobius shangchengensis* (Urodela: Hynobiidae). J. Herpetol..

[53] Nishikawa K., Jiang J.P., Matsui M., Mo Y.M., Chen X.H., Kim J.B., Tominaga A., Yoshikawa N. (2010). Invalidity of *Hynobius yunanicus* and molecular phylogeny of *Hynobius* salamander from continental China (Urodela, Hynobiidae). Zootaxa.

[54] Jin Y. (1994). The Demi-Gods and Semi-Devils.

[55] Heo K., Shin Y., Messenger K.R. (2022). *Hynobius notialis* (Southern Korean Salamander). Behavior. Herpetol. Rev..

[56] Fauth J.E., Resetarits W.J. (1999). Biting in the salamander *Siren intermedia intermedia*: Courtship component or agonistic behavior?. J. Herpetol..

[57] Zhang P., Chen Y.-Q., Zhou H., Liu Y.-F., Wang X.-L., Papenfuss T.J., Wake D.B., Qu L.-H. (2006). Phylogeny, evolution, and biogeography of Asiatic salamanders (Hynobiidae). Proc. Natl. Acad. Sci. USA.

[58] Lu B., Zheng Y., Murphy R.W., Zeng X. (2012). Coalescence patterns of endemic Tibetan species of stream salamanders (Hynobiidae: *Batrachuperus*). Mol. Ecol..

[59] Li J., Fu C., Lei G. (2011). Biogeographical consequences of Cenozoic tectonic events within East Asian margins: A case study of *Hynobius* biogeography. PLoS ONE.

[60] Zhou Y., Wang S., Zhu H., Li P., Yang B., Ma J. (2017). Phylogeny and biogeography of South Chinese brown frogs (Ranidae, Anura). PLoS ONE.

[61] Jacques F.M., Shi G., Su T., Zhou Z. (2015). A tropical forest of the middle Miocene of Fujian (SE China) reveals Sino-Indian biogeographic affinities. Rev. Palaeobot. Palynol..

[62] Che R., Sun Y., Wang R., Xu T. (2014). Transcriptomic analysis of endangered Chinese salamander: Identification of immune, sex and reproduction-related genes and genetic markers. PLoS ONE.

[63] IUCN Standards and Petitions Committee (2019). Guidelines for Using the IUCN Red List Categories and Criteria.

[64] Shin Y., Min M.S., Borzée A. (2021). Driven to the edge: Species distribution modeling of a Clawed Salamander (Hynobiidae: *Onychodactylus koreanus*) predicts range shifts and drastic decrease of suitable habitats in response to climate change. Ecol. Evol..

[65] Green D.M., Lannoo M.J., Lesbarrères D., Muths E. (2020). Amphibian population declines: 30 years of progress in confronting a complex problem. Herpetologica.

[66] Moor H., Bergamini A., Vorburger C., Holderegger R., Bühler C., Egger S., Schmidt B.R. (2022). Bending the curve: Simple but massive conservation action leads to landscape-scale recovery of amphibians. Proc. Natl. Acad. Sci. USA.

